# Rate and Temporal Coding Mechanisms in the Anterior Cingulate Cortex for Pain Anticipation

**DOI:** 10.1038/s41598-018-26518-x

**Published:** 2018-05-29

**Authors:** Louise Urien, Zhengdong Xiao, Jahrane Dale, Elizabeth P. Bauer, Zhe Chen, Jing Wang

**Affiliations:** 1 Department of Anesthesiology, Perioperative Care, and Pain Medicine, New York University School of Medicine, New York, New York 10016 USA; 2 Department of Psychiatry, New York University School of Medicine, New York, New York 10016 USA; 3 Department of Neuroscience and Physiology, New York University School of Medicine, New York, New York 10016 USA; 4 Biology Department, Barnard College Columbia University, New York, New York 10027 USA; 5 Department of Instrument Science and Technology, College of Biomedical Engineering and Instrument Science, Zhejiang University, Hangzhou, Zhejiang China

## Abstract

Pain is a complex sensory and affective experience. Through its anticipation, animals can learn to avoid pain. Much is known about passive avoidance during a painful event; however, less is known about active pain avoidance. The anterior cingulate cortex (ACC) is a critical hub for affective pain processing. However, there is currently no mechanism that links ACC activities at the cellular level with behavioral anticipation or avoidance. Here we asked whether distinct populations of neurons in the ACC can encode information for pain anticipation. We used tetrodes to record from ACC neurons during a conditioning assay to train rats to avoid pain. We found that in rats that successfully avoid acute pain episodes, neurons that responded to pain shifted their firing rates to an earlier time, whereas neurons that responded to the anticipation of pain increased their firing rates prior to noxious stimulation. Furthermore, we found a selected group of neurons that shifted their firing from a pain-tuned response to an anticipatory response. Unsupervised learning analysis of ensemble spike activity indicates that temporal spiking patterns of ACC neurons can indeed predict the onset of pain avoidance. These results suggest rate and temporal coding schemes in the ACC for pain avoidance.

## Introduction

Anticipating the likelihood of an aversive event, such as pain, is an important adaptive behavior. Anticipation of pain may lead to natural responses such as escape or avoidance, in order to minimize immediate or future physical harm. Therefore, understanding the neural substrate for pain anticipation or avoidance is important for our knowledge of pain processing in the brain.

Passive responses to pain, such as freezing, involve deep brain structures such as the amygdala, the hippocampus or the periaqueductal gray^1,2^. In contrast, cortical regions such as the prefrontal cortex may play a role in active avoidance and escape^3^. The anterior cingulate cortex (ACC) is involved in many cognitive and affective processes such as attention^4–7^, decision making^8–10^, and prediction of action and rewards^11–13^. Importantly, ACC neurons are well-known to be involved in pain processing. Electrophysiological studies have characterized changes in the firing rates of ACC neurons in response to an acute pain signal^14–18^. Meanwhile, numerous studies have indicated the role of ACC in the affective processing of pain^19–22^. In particular, the ACC has been found to process the aversive component of pain, including pain intensity^17,23–25^. Both population and rate coding schemes have been proposed in the ACC for understanding the intensity of pain signals^17,26^. In addition, it has been suggested that temporal coding of the painful stimulus in the ACC is altered in chronic pain states^27^. Finally, neuroimaging studies have shown that the ACC is activated during pain anticipation^28–32^. However, despite our knowledge of changes in the firing rates and patterns of ACC neurons in response to pain^33–36^, little is known about how ACC neurons change their firing during the period of anticipation of pain signals and how those changes might contribute to pain avoidance. We hypothesized that a specific population of neurons may encode pain anticipation and avoidance behaviors. Furthermore, as avoidance is a progressive learned behavior, time and rate firing of specific ACC neurons may also contribute to a fine-tuning of pain anticipation.

In this study, we focused on how anticipation of a painful stimulus can lead to its avoidance. We established a pain avoidance assay, and found that approximately two thirds of the rats progressively learned to avoid the noxious stimulus. We also performed *in vivo* electrophysiological recordings of ACC neurons in these awake, free-behaving rats, during the conditioning assay. These recordings allowed us to identify a number of individual ACC neurons selectively responding to the anticipation of pain, or the pain stimulus itself. We found that neurons that tended to fire during the anticipation period increased their firing rate, possibly contributing to an escape behavior, whereas neurons that typically responded to pain fired earlier in response to the noxious stimulus. Furthermore, we found specific ACC neurons that shifted their firing from a pain-tuned response to an earlier “anticipatory” response. Together, these three groups of neurons can provide important information for pain anticipation and avoidance. Finally, unsupervised machine-learning analysis of single-trial population spike data demonstrated accurate detection of the timing of pain anticipation, further confirming the rate and temporal coding mechanisms in the ACC for pain anticipation.

## Results

### Rats can learn to anticipate and consequently avoid pain

**Figure 1:**
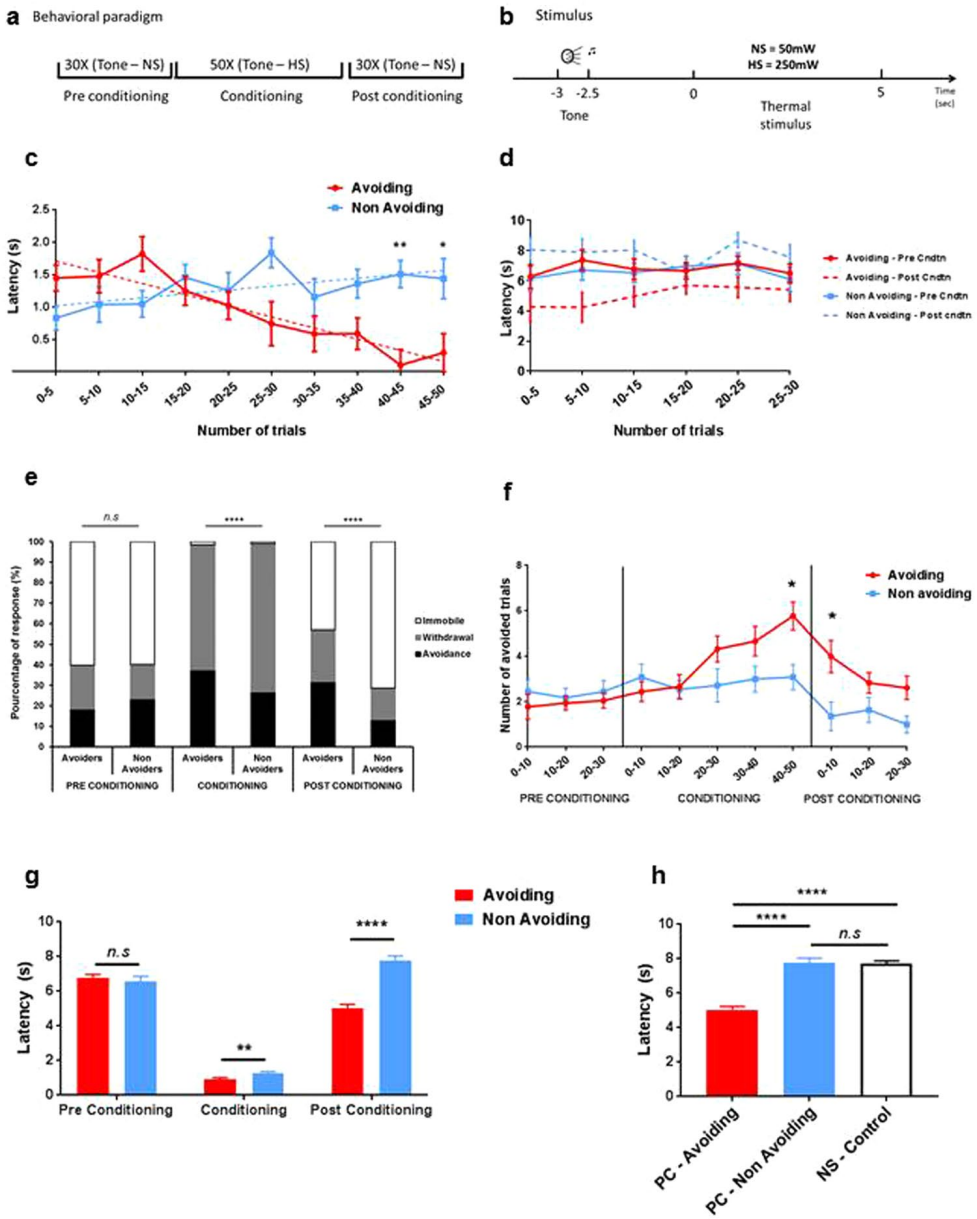
A conditioning paradigm leads rat to progressively avoid pain. (**a**) Experimental paradigm. Pre-conditioning/Post-conditioning phase: tone (4 KHz, 80 dB) paired with a non-noxious heat stimulus (NS-50 mW) repeated 30 times. Conditioning phase: tone (4 KHz, 80 dB) paired with a noxious heat stimulus (HS-250 mW). (**b**) Trial = tone delivered 3 seconds before the heat stimulus for 500 mseconds; heat stimulus applied for 10 seconds (NS), 5 seconds (HS) or until the animal withdraw its hindpaw. (**c**) Different responses during pain conditioning. The avoiding group (13/20 animals, red line) exhibits a decrease in withdrawal latency over time (y = −0.035x + 1.88) whereas the non-avoiding group exhibits no changes (y = 0.012x + 0.96). Significant differences between the 2 groups at the end of the conditioning (trials #40–45, 0.10 ± 0.24 vs 1.51 ± 0.21 sec, p = 0.003; trials #45–50, 0.29 ± 0.3 vs 1.44 ± 0.31 sec, p = 0.03, n = 18 and 11 respectively, two-way ANOVA, post-hoc Bonferroni t-test). (**d**) Mean withdrawal latency. Conditioning phase, significantly shorter withdrawal latency for the avoiding group (0.92 ± 0.09 vs 1.29 ± 0.08 sec; p = 0.003, n = 18 and 11 respectively); post-conditioning phase, maintenance of a significantly shorter withdrawal latency (5.04 ± 0.21 vs 7.98 ± 0.24 sec; p < 0.0001, n = 18 and 11 respectively). (**e**) Population distribution of the animals behavior classified according to the time of paw movement (−3 to 0 = avoidance/0 to 5–10 = withdrawal/>5–10 = immobile). Higher proportion of avoidance during conditioning, sustained during post conditioning (p < 0.0001, Chi square test, n = 18 and 11 respectively). (**f**) Number of avoided trials during the 3 phases. Increased number of avoided trials in avoiding rats during the conditioning, significantly higher than for non-avoiding rats (trials #40–50, 5.78 ± 0.61 vs 3.09 ± 0.56, p = 0.01). Maintenance during post conditioning (trials #0–10, 4 ± 0.71 vs 1.36 ± 0.64, p = 0.01) (n = 18 and 11 respectively, two-way ANOVA, post-hoc Bonferroni t-test). (**g**) Withdrawal latency over time during pre and post conditioning. No difference between the two groups during pre- conditioning. At the beginning of post-conditioning, significant differences (trials #0–5, 4.28 ± 0.98 vs 8.06 ± 0.79 sec, p = 0.024; trials #5–10, 4.24 ± 0.99 vs 7.88 ± 0.9 sec, p = 0.037; n = 18 and 11 respectively, two-way ANOVA, post-hoc Bonferroni t-test). (**h**) Mean withdrawal latency during the post-conditioning phase (PC) between avoiding, non-avoiding rats and control animals, (NS-50 mW X 30 trials – no tone). Significantly shorter withdrawal latency for the avoiding rats (5.6 ± 0.30 vs 7.86 ± 0.28 sec vs 7.72 ± 0.16 sec, n = 18,11 and 20 respectively p < 0.0001). Data are shown as mean ± SEM.

To uncover the role of ACC neurons in pain avoidance, we designed a task where animals could anticipate a painful stimulus and avoid it. We trained rats with a conditioning paradigm including three phases (Fig. 1a,b). During the pre-conditioning phase, a tone signal (4 KHz, 80 dB, 0.5 sec) was paired with a non-painful thermal stimulus (NS) applied to the rat’s hind paw. During the conditioning phase, we paired the same tone with a painful heat stimulus (HS) for a total of 50 trials to induce pain avoidance. Indeed, the rat could avoid the painful stimulus by simply removing its paw after the tone has been played, before the laser was turned ON. In this case, the noxious stimulus was not delivered. Finally, during an additional, post-conditioning phase, the tone signal was paired with NS again (Fig. 1a,b). During the 3 phases, the thermal stimulus was delivered 3 seconds after the tone was turn ON. Each trial of each animal is quantified by two factors: time of withdrawal (Fig. 1c,d,g,h) and the identity of either an ≪ avoidance ≫ or ≪ withdrawal ≫ trial (Fig. 1e,f). The two factors are linked as described hereafter. The latency of withdrawal was quantified in regard to the time of laser ON: a withdrawal occurring between laser ON and OFF was counted as a positive value and labelled a “withdrawal” trial. A withdrawal occurring after tone ON and before laser ON was counted as a negative value and labelled an “avoidance” trial and no stimulus was delivered. No withdrawal occurring during the entire trial was counted as a positive value (either 5 sec for conditioning or 10 sec for pre or post) and labelled an “immobile” trial. During the conditioning phase there was heterogeneity of behavioral responses. Over time, 13 of 20 rats progressively learned to avoid the painful stimulus by withdrawing their hind paws. These animals exhibited an overall decrease in withdrawal latency demonstrated by an inverse linear relationship with the number of trials (Fig. 1c, red line; red dashes y = −0.035x + 1.88). Thus, we called these rats “pain-avoiding” rats. In contrast, 7 of 20 rats did not exhibit this behavior. Instead, these animals exhibited no changes in withdrawal latency throughout all the trials of the conditioning phase (Fig. 1c; blue line; blue dashes y = 0.012x + 0.96). These rats were referred to as “non-avoiding” rats. Pain-avoiding rats demonstrated a shorter paw withdrawal latency compared to the non-avoiding group (avoiding group 0.92 ± 0.1 sec; non-avoiding group, 1.29 ± 0.08 sec; p = 0.034, n = 18 and 11 respectively, unpaired t-test, Fig. 1d) with a particular significance at the end of the conditioning phase (trials #40–45, p = 0.003; trials #45–50, p = 0.032; n = 18 and 11 respectively, two-way ANOVA followed by post-hoc Bonferroni t-test, Fig. 1c). This difference in paw withdrawal latency is based on a higher proportion of avoided trials in the avoiding group during conditioning (avoiding rats 37.59 ± 4.56% vs non-avoiding rats 26.1 ± 4.38%; p < 0.0001, chi square test, Fig. 1e; trials #40–50, 5.78 ± 0.61 vs 3.09 ± 0.56, p = 0.01, n = 18 and 11 respectively, two-way ANOVA, post-hoc Bonferroni t-test, Fig. 1f). This distinction of avoiding and non-avoiding subjects mirrors the finding from previous studies^37–39^.

Next, we examined the short-term stability of this pain avoiding behavior. We found that in addition to demonstrating a shorter latency to withdrawal time during the conditioning phase, rats in the avoiding group also demonstrated decreased latency to withdrawal during the post-conditioning phase (PC) (Fig. 1d), specifically at the beginning of the conditioning phase (trials #0–5, avoiding group: 4.28 ± 0.98 sec, non-avoiding group 8.06 ± 0.79 sec, p = 0.024; trials #5–10, avoiding group 4.24 ± 0.99 sec, non-avoiding group 7.88 ± 0.9 sec, p = 0.037; n = 18 and 11 respectively, two-way ANOVA followed by post-hoc Bonferroni t-test, Fig. 1g). Again this difference was supported by a higher number of avoided trials in the avoiding group (avoiding rats 31.93 ± 5.01% vs non-avoiding rats 13.27 ± 4.46%; p < 0.0001, chi square test, Fig. 1e; trials #0–10, 4 ± 0.71 vs 1.36 ± 0.64, p = 0.01, n = 18 and 11 respectively, two-way ANOVA, post-hoc Bonferroni t-test, Fig. 1f). This behavior was not seen in non-avoiding rats, nor rats that did not undergo this conditioning protocol (PC avoiding group 5.04 ± 0.21 sec; PC non-avoiding group, 7.78 ± 0.24 sec; NS-Control, 7.72 ± 0.16 sec, p < 0.0001, n = 18,11 and 20 respectively, unpaired t-test, Fig. 1g,h). These data during the conditioning and post-conditioning phases demonstrate that this conditioning paradigm allowed us to identify rats that anticipated and avoided pain and rats that did not.

### Specific rate and time coding schemes in individual ACC neurons for pain anticipation

**Figure 2:**
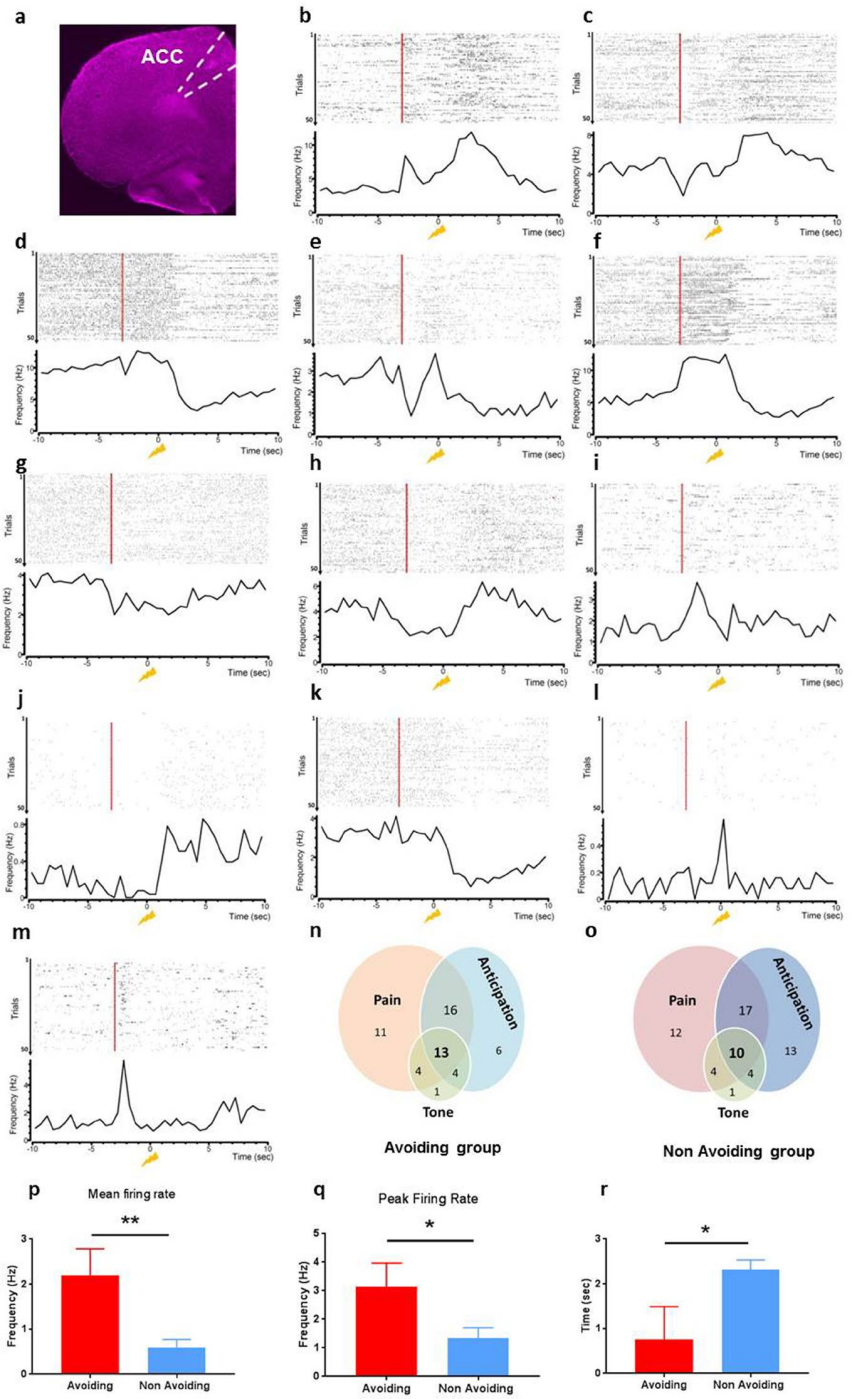
Pain avoidance does not require recruitment of specific neurons. (**a**) Histology of the location of recording tetrodes in the ACC. (**b**–**m**) Response of individual ACC neurons. Raster plots (top) and PSTHs (bottom) of 12 representative neurons (12 of 154) aligned to the time of the onset of the pain stimulus (yellow strike). Red lines indicate the onset of the tone. (**n**–**o**) Venn diagram of overlap between subpopulations that significantly modulate their firing rate (increased or decreased) during 3 time periods: tone (−3 to −2.5 sec), anticipation (−2.5 to 0 sec) and pain (0 to 3.5 sec). See Methods for details. Fisher’s exact tests demonstrate relatively equal distribution of ACC response between the two group of rats (data not shown). (**p**) Mean firing rate of neurons that increased their spiking after the pain stimulus (“pain-anticipating” neurons, see Methods for details) is higher in the avoiding group than non-avoiding group (avoiding group 2.2 ± 0.59 Hz, non-avoiding group 0.6 ± 0.17 Hz, n = 6 and n = 11 respectively, p = 0.027). (**q**) Peak firing rate of “pain-anticipating” neurons is higher in the avoiding group than non-avoiding group (avoiding group 3.14 ± 0.83 Hz, non-avoiding group 1.34 ± 0.37 Hz, n = 6 and n = 11 respectively, p = 0.039). (**r**) Time of the peak firing rate of “pain responsive” neurons is earlier in the avoiding group than non-avoiding group (avoiding group 0.75 ± 0.74 sec, non-avoiding group 2.32 ± 0.22 sec, n = 8 and n = 11 respectively, p = 0.032). Data are shown as mean ± SEM.

To define a neural substrate for pain avoidance, we recorded the activity of neural ensembles in the ACC during the behavioral task outlined above, using chronically implanted custom tetrodes. A total of 154 single ACC units were recorded in 9 animals (Fig. 2a). We compared the neuronal activity between the avoiding group (5/9 rats, 72 neurons) and the non-avoiding group (4/9 rats, 82 neurons). First, we hypothesized that in order for a rat to avoid a painful stimulus, the animal should be able to anticipate it. Previous studies have shown that within the ACC, a significant portion of neurons increase their firing rates specifically in response to acute pain stimuli^14–18^. Here, we tested the possibility that a specific group of ACC neurons were activated before pain stimulation. These “pain-anticipating” neurons would increase or decrease their spike rates after the tone, and prior to, or in anticipation of, the pain stimulus. We also investigated the possibility that distinct groups of neurons could be recruited in the avoiding and non-avoiding rats, which might contribute to behavior. Hence, in the pain-avoiding group, activation of “pain-anticipating” neurons could provide the signals for pain avoidance. A lack of activation of those neurons in non-avoiding animals, on the other hand, will explain the non-avoidance behavior.

We thus examined the temporal response profiles during different phases of learning by plotting spike rasters and peri-event time histograms (PETHs) of individual neurons, relative to the onset of the noxious stimulus. During the conditioning phase, we observed multiple phenotypes. Many neurons increased or decreased their firing rates from baseline at different time points (Fig. 2b–h). Although some neurons responded exclusively to the pain stimulus (0 to 3.5 sec; “pain-responsive” neurons (see Materials and Methods^17^) (Fig. 2j,k), many ACC neurons also fired at other distinct time points during the conditioning phase e.g. when the tone was played (−3 to −2.5 sec) (Fig. 2m). We observed both transient (Fig. 2b,c,e,i) and sustained (Fig. 2f) responses to the tone or to the pain stimulus (Fig. 2j,k). Finally, we found ACC neurons that changed their firing rates only during the period between the tone and pain stimulus, specifically during the conditioning phase (we term this the anticipation period) (Figs 2l and S1).We characterized those neurons that specifically changed their firing rates during the anticipation period as “pain-anticipating” neurons. We first asked whether those specific neurons where differentially activated during withdrawal versus avoided trials. We found some examples of neurons preferentially activated during the “anticipation period” during the avoided trials, some example of neurons increasing their firing rate after the painful stimulus during the withdrawal trials, and neurons presenting both activation patterns (Fig. S2). However because of the heterogeneity of the behavioral responses, we were not able to provide statistical analysis. We then compared the number of “pain-anticipating” neurons in the avoiding and the non-avoiding rats. In the avoiding rats, 30.55% (22 of 72) of the neuronal population demonstrated significantly altered firing rates during the tone, 54% (39 of 72) of the neurons changed their firing rates during the anticipation period and 61% (44 of 72) of the neurons changed their firing rates after the pain stimulus (Fig. 2n). Detailed analysis revealed that 15.3% (11 of 72) of recorded neurons only responded (by increasing or decreasing their firing rates) to the painful stimulus, 1.4% (1 of 72) neurons only responded during the tone (−3 to −2.5 sec), and 8.3% (6 of 72) changed their firing rates only during the anticipation period. In the non-avoiding group, however, we found approximately the same proportion and pattern of responsive neurons for each time period (Fig. 2o – Fisher exact test – non significant, data not shown). These comparable distributions have several implications. First, they suggest that the coding of pain avoidance does not necessarily require the recruitment of a distinct specific group of neurons within the ACC. Second, the activation of “pain-anticipating” neurons in both group of animals suggest that both groups may anticipate pain. Finally, activation of “pain-anticipating” neurons within the ACC alone is likely not sufficient to produce pain avoidance.

If the coding of pain avoidance does not rely on a specific neuronal activation of “pain-anticipating” neurons within the ACC, another possibility is a time or rate modulation of their neuronal activity. Indeed, during the conditioning phase, with progressive trials where a tone was played before the noxious stimulus, some rats exhibited a gradual earlier shift in the onset of the withdrawal time. This shift in the time domain suggests the possibility of a temporal coding scheme. We examined if a time coding scheme is manifested at the level of individual neurons, especially within the distinct subpopulation we described earlier, “pain-anticipating” neurons and “pain-responsive” neurons. We investigated the likelihood of two possible mechanisms that could contribute to a time coding paradigm. The first possible mechanism is that “pain-anticipating” and “pain-responsive” neurons are successively recruited; the second potential mechanism is that the same neurons that normally respond to pain stimuli shifted their firing responses earlier in the conditioning phase.

We examined the changes in the firing rate and the timing in individual ACC neurons by comparing the mean and peak firing rates, as well as the onset of peak firing rates for these “pain-anticipating” and “pain-responsive” neurons. We first examined the firing rates of the “pain-anticipating” neurons during the pain anticipation period. We found that neurons from the avoiding group demonstrated a higher mean and peak firing rate than similar neurons from the non-avoiding group (mean firing rate: 2.2 ± 0.59 Hz vs 0.6 ± 0.17 Hz, n = 6 and n = 11 respectively, p = 0.027, Mann-Whitney U-test, Fig. 2p; peak firing rate: 3.14 ± 0.83 Hz vs 1.34 ± 0.37 Hz, n = 6 and n = 11 respectively, p = 0.039, Mann-Whitney U-test, Fig. 2q).

We then turned our attention to “pain-responsive” neurons. These neurons are defined as cells that increased or decreased their firing rates after the pain stimulus^17^. “Pain-responsive” neurons in the avoiding rats reached peak firing rates sooner after the onset of pain stimulus compared to neurons from non-avoiding rats (0.75 ± 0.74 sec vs 2.32 ± 0.22 sec, n = 8 and n = 11 respectively, p = 0.032, Fig. 2r). These results demonstrate a temporal coding scheme for pain responsiveness in these neurons as well.

These results of neural analysis are compatible with the behavior data that showed shorter paw withdrawal latency for pain-avoiding rats (Fig. 1d). Together, these data suggest that pain avoidance does not depend on the recruitment of a specific group of ACC neurons, but rather on the rate and timing of two different groups of neurons.

### A specific group of neurons provide additional time coding for pain avoidance

**Figure 3:**
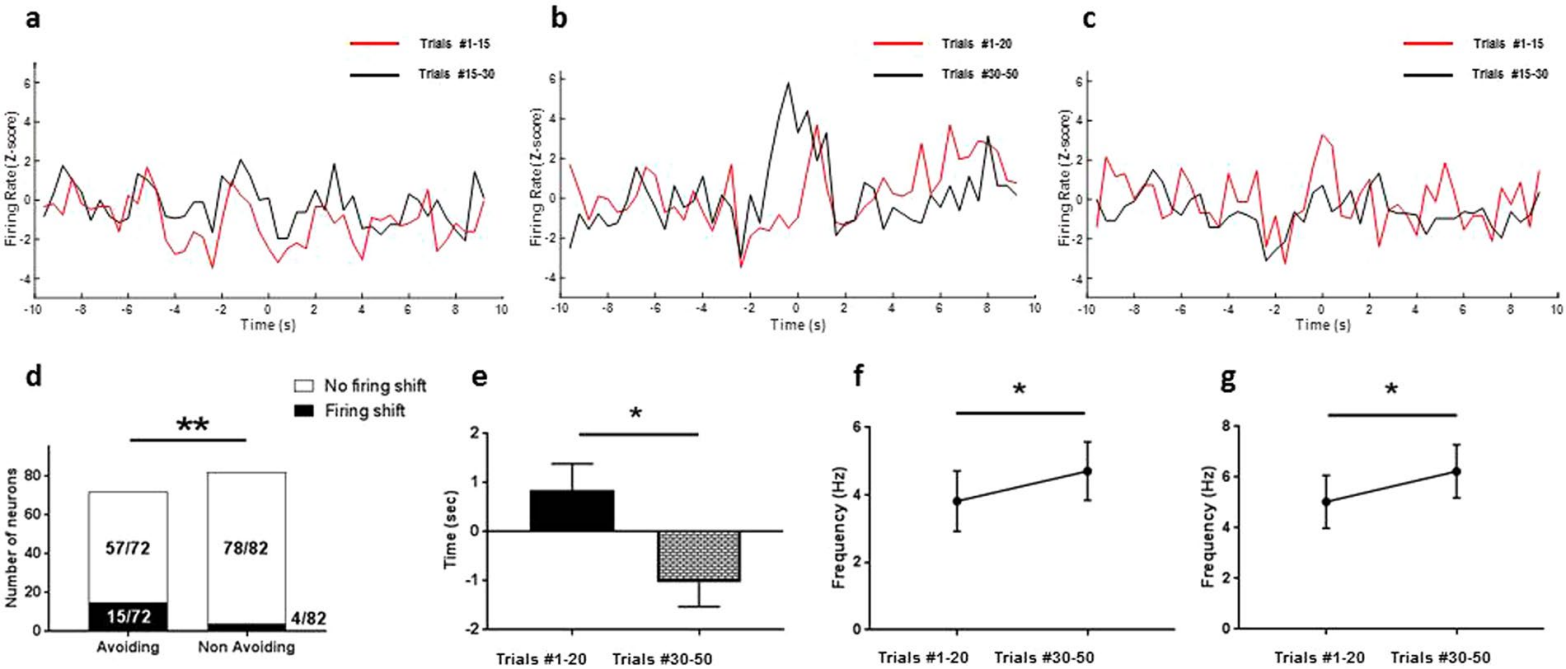
Specific ACC neurons shift the timing of their peak firing to encode pain avoidance. (**a**–**c**) Firing rate of a representative neuron that shifted its peak firing rate to earlier time point around the painful stimulus. Time = 0 corresponds to the time of the onset of the pain stimulus. (**a**) Pre-conditioning, trials #1–15 (red line); trials #15–30 (black line). (**b**) Conditioning, trials #1–20 (red line); trials #30–50 (black line); this example neuron shifted its timing of peak firing from after pain stimulus (red line) to before pain stimulus (dark line). (**c**) Post-conditioning, trials #1–15 (red line); trials #15–30 (black line). (**d**) Histogram compiling the number of neurons presenting a shift in firing peak during conditioning (black bar). 15/72 neurons in the avoiding group (left histogram) demonstrate this shift in timing. In contrast, only 4/82 in the non-avoiding group (right histogram) showed this phenotype (p = 0.031, Fisher’s exact test). (**e**) Time of peak firing of neurons in *d* is earlier at the beginning of the conditioning period than the end (trials #1–20: 1.25 ± 0.52 seconds; trials #30–50: −0.73 ± 0.52 seconds; n = 13; p = 0.0193). (**f**) Mean firing rate of these neurons that shift the timing of their peak spiking response during the tone is significantly higher at the beginning than at the end of the conditioning (3.82 ± 0.9 Hz vs 4.71 ± 0.9 Hz, n = 13, p = 0.034). (**g**) Peak firing rate of neurons that shift the timing of their peak spiking response during the tone is significantly higher at the beginning than at the end of the conditioning (4.43 ± 0.9 Hz vs 5.59 ± 1 Hz, n = 13, p = 0.04). Data are shown as mean ± SEM.

Our analysis of the ensemble neural response suggests that the timing of spikes for certain neurons could carry an important signal for pain avoidance. We next turned our attention to neurons that spiked after the pain stimulus at the beginning of the conditioning phase (trials #1–20, red line, Fig. 3b), but spiked prior to the pain stimulus during the later trials of the conditioning phase (trials #30–50, black line, Fig. 3b) (trials #1–20: 1.25 ± 0.52 seconds; trials #30–50: −0.73 ± 0.52 seconds; n = 13; p = 0.0193, Wilcoxon matched-pairs signed rank test, Fig. 3e). We found significantly more neurons that shifted their firing rates to an earlier time point prior to pain stimulation in the pain-avoiding rats (15 of 72 neurons) than the non-avoiding group (4 of 82 neurons, Fisher exact test, p = 0.0031, Fig. 3d). These neurons that shifted the timing of their firing rates (15/72) were not the same as “pain-responsive” neurons described earlier (6/72, Fig. 2). Unlike “pain-responsive” neurons that progressively responded more quickly to the pain stimulus, these neurons stopped responding to pain and started to respond during the anticipatory period. Moreover, during the conditioning phase, these neurons exhibited a significantly increased mean firing rate during the tone presentation (4.71 ± 0.9 Hz vs 3.82 ± 0.9 Hz n = 13, p = 0.034, Wilcoxon-matched-pairs signed rank test, Fig. 3f), and an increased peak firing rate during the anticipation period (4.43 ± 0.9 Hz vs 5.59 ± 1 Hz, n = 13, p = 0.04, Wilcoxon-matched-pairs signed rank test, Fig. 3g). These results suggest that a distinct group of neurons dramatically alters the timing of their spikes as conditioning progresses. Finally, we examined these neurons during the post-conditioning phase, when rats were not presented with the noxious stimulus. Interestingly, we found that 4 of the 13 neurons in the pain-avoiding group that shifted their firing rates to an earlier time point maintained an increased firing rates during the anticipatory period at the beginning of the post-conditioning phase (trials #1–15) (red line, Fig. 3c). However, over time, these neurons resumed their firing pattern observed during the pre-conditioning phase (Fig. 3a,c).

### Pain avoidance is encoded by ensemble neural activities in the ACC

Finally, we examined if the rate and time coding described for “pain-anticipating” and “pain-responsive neurons” was reflected in the ensemble neural activities in the ACC. We examined if the amplitude and/or the frequency of neuronal responses differed between the two groups of animals. We compared the mean firing rate, peak firing rate and the timing of peak firing during the entire conditioning phase. However, we didn’t find any significant differences (Table 1). These data suggest that pain avoidance is not likely to be encoded by changes in spike rates at the general population level in the ACC but rather by discrete neurons, as described previously.

**Table 1:** Firing rate analysis during conditioning.

		Conditioning				
		Baseline (−*9 to* −*6 sec*)	Tone (−*3 to* −*2*.*5* *sec*)	Anticipation (−*2*.*5 to 0* *sec*)	Pain (*0 to 3*.*5* *sec*)	Total (−*3 to 3*.*5* *sec*)
*Anticipating*	Mean firing rate (Hz)	2.717 ± 0.4721	2.789 ± 0.4184	2.878 ± 0.4499	2.582 ± 0.487	2.71 ± 0.516
	Peak firing rate (Hz)	3.09 ± 0.5	2.95 ± 0.48	3.34 ± 0.50	3.46 ± 0.64	3.95 ± 0.67
	Time of peak firing rate (Hz)					−0.24 ± 0.23
*Non Anticipating*	Mean firing rate (Hz)	2.507 ± 0.341	2.519 ± 0.3643	2.323 ± 0.3311	2.1 ± 0.28	2.1 ± 0.28
	Peak firing rate (Hz)	2.70 ± 0.34	2.50 ± 0.42	3.12 ± 0.4	2.78 ± 0.36	3.6 ± 0.48
	Time of peak firing rate (Hz)					−0.56 ± 0.21
*Statistical significances Anticipating vs Non Anticipating*	Mean firing rate (Hz)	p = 0.91	p = 0.93	p = 0.54	p = 0.91	p = 0.61
	Peak firing rate (Hz)	p = 0.63	p = 0.58	p = 0.99	p = 0.49	p = 0.84
	Time peak firing rate (sec)					p = 0.31

Mean firing rate, peak firing rate and time of the peak firing rate for neuronal population of avoiding and non-avoiding groups during conditioning. Statistical significances are calculated using Mann-Whitney U-test for frequency and unpaired t-test for time.

**Figure 4:**
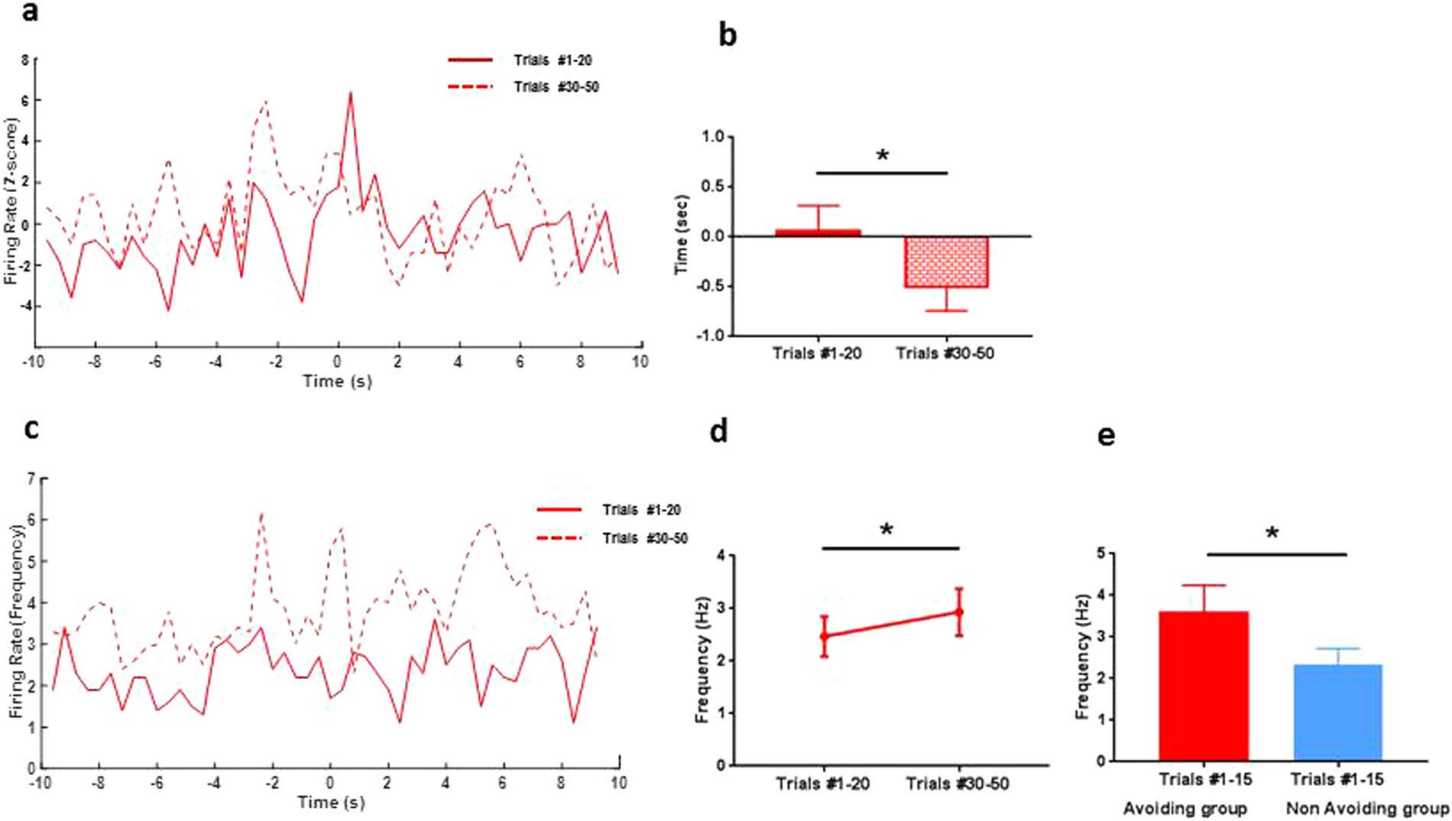
Pain avoidance is encoded by ensemble neural activities in the ACC. (**a**) Response of representative ACC neuron shifting its peak firing rate from a late response during the first trials (red line) to an earlier during the last trials (dashed red line). (**b**) Time of the peak firing rate of the neuronal population in the avoiding group decreasing between the beginning (trials #1–20) and the end (trials #30–50) of the conditioning phase (trials #1–20, 0.076 ± 0.24 sec, trials #30–50, −0.51 ± 0.23 sec n = 72, p = 0.0482). (**c**) Response of a representative ACC neuron increasing its peak firing rate to tone signal during the last trials (dashed red line) compared to the first trials (red line). (**d**) Increase of the mean firing rate, during the tone, of ACC neurons in the avoiding group between the beginning (trials #1–20) and the end (trials #30–50) of the conditioning phase (trials #1–20, 2.467 ± 0.38 Hz, trials #30–50, 2.93 ± 0.45, n = 72, p = 0.01). (**e**) Higher peak firing rate, during the tone presentation, in the avoiding group compared to the non-avoiding group at the beginning of the post-conditioning (trials #1–15) (avoiding group, 3.61 ± 0.63 Hz; non-avoiding group, 2.35 ± 0.37, n = 72 and 82 respectively, p = 0.0351). Data are shown as mean ± SEM.

Thus, we compared the ensemble neuronal activity between the first 20 trials (#1–20) of the conditioning phase (during which the rats did not clearly avoid pain) with the last 20 trials (#30–50) of this same phase (during which pain-avoiding rats acquired the ability to avoid pain). We found that, for pain-avoiding rats, the time of peak firing occurred significantly earlier at the end of the conditioning phase (Fig. 4a – dashed red line) compared to the beginning of it (Fig. 4a – red line) (trials #1–20, 0.076 ± 0.24 sec; trials #30–50, −0.51 ± 0.23 sec, n = 72, p = 0.0482, Wilcoxon matched-pairs signed rank test, Fig. 4b). This data indicates that during the later trials, many of the ACC neurons began to fire before the onset of the pain stimulus. In addition, neurons of the avoiding rats also demonstrated a higher firing rate in response to the tone stimulus during the later trials (Fig. 4c – dashed red line) compared to earlier trials (Fig. 4c – red line) during the conditioning period (trials #1–20, 2.467 ± 0.38 Hz, trials #30–50, 2.93 ± 0.45, n = 72, p = 0.01, Wilcoxon matched-pairs signed rank test, Fig. 4d). These results indicate that during conditioning, when the rats repeatedly associated a neutral stimulus – tone – with a painful stimulus, their neurons in the ACC progressively responded with increased amplitude to the neutral stimulus.

To verify the stability of this temporal coding scheme for the pain avoidance behavior, we examined the firing pattern of the ACC neurons during the post-conditioning phase. We compared ensemble neural activities during the first 15 trials (trials #1–15) of the post-conditioning phase with activities during the last 15 trials (trials #15–30). To our surprise, we did not detect any statistically significant difference in the mean firing rate, peak firing rate, and the time of peak firing rate (Table 2). In contrast, we found that at the beginning of the post-conditioning phase, the peak firing rate during the tone stimulus was higher in the pain-avoiding group (avoiding group, 3.61 ± 0.63 Hz; non-avoiding group, 2.35 ± 0.37, n = 72 and 82 respectively, p = 0.0351, Mann-Whitney U-test, Fig. 4e). These results suggest that during conditioning, ACC neurons begin to develop an increase in the responsiveness to the neutral stimulus (tone), and this increase was maintained at the beginning of the post-conditioning phase. Later in the post-conditioning phase, however, ACC neurons returned to their baseline behaviors. This return is expected as the tone stimulus was no longer paired with a pain stimulus.

**Table 2:** Firing rate analysis during post conditioning.

		Post Conditioning # 1–15					Post conditioning # 15–30				
		Baseline (*−9 to* −*6 sec*)	Tone (−*3 to* −*2*.*5* *sec*)	Anticipation (−*2*.*5 to 0* *sec*)	Pain (*0 to 3*.*5* *sec*)	Total (−*3 to 3*.*5* *sec*)	Baseline (−*9 to* −*6 sec*)	Tone (−*3 to* −*2*.*5* *sec*)	Anticipation (−*2*.*5 to 0* *sec*)	Pain (*0 to 3*.*5* *sec*)	Total (−*3 to 3*.*5* *sec*)
*Anticipating*	Mean firing rate (Hz)	2.71 ± 0.48	2.64 ± 0.47	2.98 ± 0.56	2.97 ± 0.57	2.97 ± 0.57	2.76 ± 0.57	2.76 ± 0.55	2.97 ± 0.6	2.85 ± 0.55	2.85 ± 0.55
	Peak firing rate (Hz)	4.03 ± 0.6	3.61 ± 0.63	4.37 ± 0.64	4.03 ± 0.62	4.84 ± 0.7	4.01 ± 0.6	3.9 ± 0.61	4.29 ± 0.68	4.33 ± 0.65	4.82 ± 0.7
	Time of peak firing rate (Hz)					−0.75 ± 0.23					−0.32 ± 0.25
*Non Anticipating*	Mean firing rate (Hz)	1.85 ± 0.26	1.99 ± 0.29	1.99 ± 0.29	2.17 ± 0.32	2.17 ± 0.32	2.21 ± 0.38	2.22 ± 0.38	2.18 ± 0.32	2.07 ± 0.34	2.07 ± 0.34
	Peak firing rate (Hz)	3.15 ± 0.38	2.35 ± 0.37	3.32 ± 0.42	3.09 ± 0.37	3.69 ± 0.46	3.1 ± 0.37	3.24 ± 0.33	3.44 ± 0.41	3.31 ± 0.39	3.75 ± 0.43
	Time of peak firing rate (Hz)					−0.49 ± 0.21					−0.34 ± 0.21
*Statistical significances Anticipating vs Non Anticipating*	Mean firing rate (Hz)	p = 0.06	p = 0.13	p = 0.22	p = 0.31	p = 0.31	p = 0.21	p = 0.32	p = 0.43	p = 0.13	p = 0.13
	Peak firing rate (Hz)	p = 0.10	p = 0.04	p = 0.18	p = 0.20	p = 0.14	p = 0.14	p = 0.06	p = 0.34	p = 0.22	p = 0.17
	Time peak firing rate (sec)					p = 0.40					
		**Anticipating**					**Non anticipating**				
*Statistical significances #1*–*15 vs 15*–*30*	Mean firing rate (Hz)	p = 0.55	p = 0.79	p = 0.82	p = 0.82	p = 0.82	p = 0.49	p = 0.19	p = 0.018	p = 0.42	p = 0.42
	Peak firing rate (Hz)	p = 0.56	p = 0.17	p = 0.57	p = 0.06	p = 0.88	p = 0.89	p = 0.82	p = 0.52	p = 0.13	p = 0.57
	Time peak firing rate (sec)					p = 0.17					p = 0.62

Mean firing rate, peak firing rate and time of the peak firing rate for neuronal population of avoiding and non-avoiding groups during post conditioning. Statistical significances are calculated using Mann-Whitney U-test for frequency and unpaired t-test for time when comparing anticipating versus non anticipating groups; Wilcoxon matched-pairs signed rank test frequency and paired t-test for time when comparing trials #1–15 versus trials #15–30 in one group.

**Figure 5:**
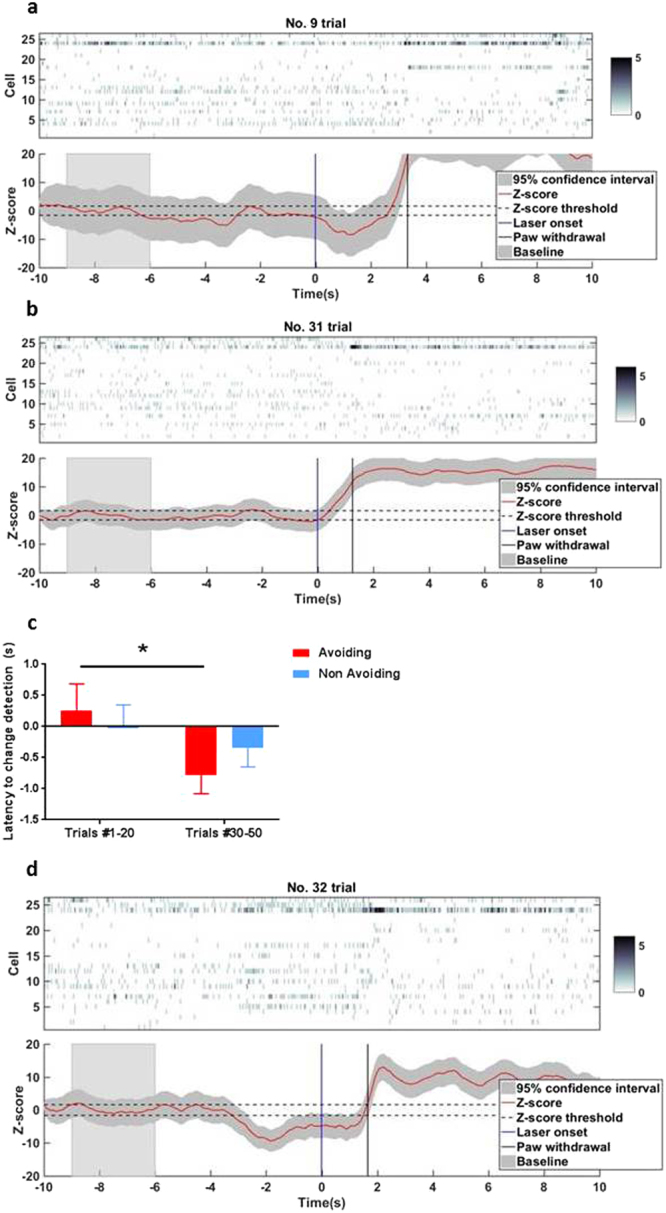
Unsupervised learning method detects a change point in population spike activity earlier at the end of the conditioning within the anticipating group. Single-trial decoding analysis in an ACC neuronal population (26 units) from a rat of the avoiding group. (**a**,**b**,**d**) Sorted population spike counts (top panel). Bin size 50 ms. Color bar indicates spike count, with the dark color representing high spike count. Bottom panel: estimated mean Z-score (red curve) from the univariate latent state. The shaded area marks the confidence intervals. Baseline is calculated from −9 to −6 sec (grey bar). The vertical blue lines indicate the laser onset (time = 0 sec), vertical black lines the paw withdrawal. Horizontal dashed lines mark the thresholds of the significant zone. (**a**) Example of one trial at the beginning of the conditioning phase (trial #9). (**b**) Example of one trial at the end of the conditioning phase (trial #31). The model-based method (Materials and Methods) can detect a change in population spike activity ~3 sec after laser onset (and 0.5 s before paw withdrawal) on trial #9 then earlier (~1 sec after the laser onset) on trial #31. (**c**) Histograms of the latency when the method detects a significant change in firing rate (mean ± SEM) of the neuronal population within the avoiding group decreasing between the beginning (trials #1–20) and the end (trials #30–50) during the conditioning phase (trials #1–20, 0.25 ± 0.43 sec; trials #30–50, −0.78 ± 0.3, n = 6 sessions/66 neurons, p = 0.03, rank-sum test). (**d**) Example of one trial at the end of the conditioning phase (trial #32). Our statistical method can also detect a change in population spike activity in response to the tone (−2 sec).

To confirm this temporal coding mechanism at the population level, we turned to an unbiased assessment of ACC ensemble activities using an unsupervised machine learning analysis. We applied a state-space model to detect the change in population spike activity of ACC neurons (see Materials and Methods). This model has previously been used to decode the onset of pain with high sensitivity and specificity^18,40–42^. Here, we analyzed single-trial ACC population spike activities during conditioning trials, and extracted the time at which the state-space model detects a significant change in population firing in relation to the baseline. We tried to compare the detected change latency between avoided and withdrawal trials (Fig. S3). However as mentioned earlier, because of an uneven number of trials in each category we were not able to provide a statistical analysis. We then compared the detected change latency between the avoiding and non-avoiding groups. We found that there was a statistically significant change in the neural ensemble activity at the end of the conditioning phase (trials #30–50, Fig. 5b) compared to the beginning (trials #1–20, Fig. 5a) for the avoiding group (change-point detection latency: trials #1–20, 0.511 ± 0.390 sec; trials #15–30, −0.954 ± 0.275 sec, n = 6 sessions/66 neurons, p = 0.03, rank-sum test, Fig. 5c). In contrast, we did not find such changes in the neural ensemble activity of non-avoiding rats (Fig. 5c). Furthermore, as shown in an example trial in Fig. 5d, our statistical method also detected the change of population activity in response to tone signals. This is consistent with our observation of higher peak firing rates of neurons during tone presentation in the avoiding group (Fig. 5d). Therefore, our unbiased population analysis using machine-learning supported the shift in time coding observed in the ACC neurons of the pain-avoiding group, as well as their increased response to the tone.

## Discussion

In this study, we asked if a selected group of neurons within the ACC could encode pain anticipation, contributing to avoidance behaviors. Furthermore, since pain avoidance is a progressive, learned behavior, we asked if temporal and rate coding schemes could also contribute to pain anticipation. We showed that after a period of conditioning, about two thirds of rats can learn to avoid pain. We identified “pain-anticipating” neurons, which exclusively spiked before the painful stimulus and “pain-responsive” neurons, which fired after the pain stimulus^17^. Activation of “pain-anticipating” neurons is not sufficient to induce pain avoidance, as these neurons were active in animals that did not learn to avoid the noxious stimulus. Instead, pain avoidance may depend on a combination of two factors. First, a select population of “pain-anticipating” neurons is recruited to anticipate pain. The higher the firing rates these neurons achieve, the greater the anticipation signal they carry allowing animals to escape. Second, “pain-responsive” neurons, which typically fire in response to the noxious stimulus, now demonstrate a faster response after the onset of the stimulus. Moreover, pain-avoiding animals possess a group of specific neurons that progressively shifted their peak firing; initially these neurons responded to pain, later these neurons fired in anticipation of the pain stimulus. Together, these three groups of neurons likely provide information for pain avoidance.

At the population level, we looked at the time of the peak firing rate of ACC neurons of those animals that learned to avoid pain, and we also found a shift towards an earlier time prior to the onset of pain stimulus during the conditioning phase. These results suggest a temporal coding scheme for pain avoidance.

There are three important concepts developed in our study. First, conditioning to a painful stimulus did not generate pain avoidance in all animals. The pain-avoiding rats in our study exhibited a decrease in paw withdrawal latency during both conditioning and post-conditioning periods. On the other hand, a group of non-avoiding rats did not demonstrate this behavioral modification. Such differences have been reported in rodents^37–39^ and humans as well^43^. The ACC neurons are known to encode the sensory averseness of a painful stimulus. Since we found the same number of “pain responsive” neurons in pain-avoiding and non-avoiding rats, and similar firing rates for these neurons, the averseness to the painful stimulus is likely comparable in both groups. Our results rather suggest the possibility that the ACC response to the neutral stimulus – tone – could play a role in pain avoidance. There could be a difference in the attention to the tone^44^, or possibly even an aversive response to the tone itself after a period of conditioning^45^. For example, some neurons responded to the tone with sustained elevation of firing rates. In particular, ACC neurons in the pain-avoiding group exhibited a higher peak firing rate than neurons in the non-avoiding group after the tone signal, during the conditioning and post-conditioning phases of the behavior paradigm. Thus, it is possible that in the pain-avoiding group, the tone over time has acquired an aversive value due to its association with pain signals. In contrast, ACC neurons in the non-avoiding rats did not acquire this association. Interestingly, some studies have shown that a neutral stimulus that is presented in temporal and/or spatial proximity to a nociceptive stimulus, can also acquire the capacity to elicit analgesia^46^. Therefore, the responsiveness of ACC neurons to the neutral learning signal may play a role in the ability to distinguish, anticipate and avoid pain. This responsiveness is likely, in turn, based on the ability of ACC neurons to perform functions in attention, prediction, and associative aversive learning^11,23,47,48^. Finally, because there was heterogeneity of behavioral responses, with an unequal number of avoided versus withdrawal trials, we were not able to conduct statistical analysis to link a specific behavioral output to a neuronal signature. However, we found examples of neurons differentially firing during avoided or withdrawal trials, and examples of single-trial decoding analysis able to detect changes in population spike activity ~2 sec before the laser onset, at the time when the animal avoided the laser stimulus. In the future, more refined behavioral tests with more segregate behavioral outputs may provide a detailed answer to this question.

Second, in our study, many individual ACC neurons responded to a combination of conditioning cues, including tone, anticipation and/or pain. Such multi-tasking illustrates a high level of diversity and complexity of information processing in the ACC^49–52^. The “pain-anticipating” neurons in our study has also been described by Koyama *et al*., as “pain anticipating responders”^34^. We have shown that their increase in firing rates is specific to pain-avoiding animals and thus may contribute to the avoidance of pain. In addition, we found “pain-responsive” neurons^17^ that shifted their spiking to an earlier time in the avoiding animals. Together, the increase in firing rates of “pain-anticipating” neurons and the earlier firing of “pain-responsive” neurons, suggest a complex pattern of activities within the ACC that encode pain anticipation and avoidance. Interestingly, it has been described that learning and conditioning can induce disinhibition of several cortical areas^53^. For example, behavioral tasks such as fear conditioning and go/no-go choice paradigm rely on the activation of local interneurons to disinhibit pyramidal neurons^54,55^. Thus, conditioning to a painful stimulus could also lead to disinhibition of the ACC pyramidal neurons, which could explain their increase in firing rates, as well as the temporal shift in spiking rates we observed in this study. Unfortunately, as we did not genetically target interneurons, we were not able to directly observe their behaviors in our conditioning paradigm. Future studies employing genetic tagging should be helpful to elucidate the role of cortical disinhibition in pain avoidance. Notably, a recent study by Schwartz and collaborators highlights the involvement of infralimbic region of PFC but not ACC in Pain Predictive Cue (PPC) avoidance paradigm in trained animals. Our results, meanwhile, showed a progressive difference in rate and time coding of avoidance during the training phase between two groups of animals. These differences may result from the use of different behavior paradigms. In the PPC avoidance paradigm, the animal is presented with a reward and a non-escapable aversive noxious stimulus that disrupts the receipt of this reward. However, in our study, animals are only presented with an aversive learning signal that the animal can actively avoid by removing its paw. Furthermore, there is active long term learning associated with the Schwartz’s study, as the authors recorded from animals that demonstrate pain avoidance 6 days after the training. We, on the other hand, recorded during the real-time behavior-acquisition period, in an acute experiment (90 min), and compared the phenotypes of avoiders versus non-avoiders animals. It is plausible that during the training period in our study, the ACC is involved in pain anticipation and its avoidance. On the other hand, this network can be further modified and stabilized, when the animals reach maximum avoidance and training, a process that requires the infralimbic cortex.

Finally, we found an interesting group of neurons that shifted their firing to an earlier time, progressively from a pain response to a pain-anticipating response. This shift mirrors what has been described in the literature for dopaminergic neurons during Pavlovian conditioning, involving an unconditioned stimulus (US) coupled with a conditioned stimulus (CS). For example, brief, phasic bursts of dopamine firing have been initially observed at the onset of US. After repeated trials, however, dopamine bursts are elicited after the onset of the CS, whereas response to the US becomes gradually attenuated. In some studies, by the end of training, US-associated dopamine signals can be effectively transferred to the CS^56^. Dopaminergic neurons have been described to have a critical role for aversive conditioning^57^. The ACC, meanwhile, receives projections from various subcortical regions, including the ventral tegmental area (VTA)^58,59^ and nucleus accumbens (NAc)^60^. These projections may modulate the firing properties of ACC neurons via D1 receptor-type dopamine signaling^61–64^. Thus, it is interesting to speculate that during conditioning, dopaminergic inputs act on specific groups of ACC neurons, producing the time coding mechanism for pain anticipation and avoidance.

It should be noted that in humans, anticipation may induce a large range of behaviors, including escaping from the pain stimulus, avoiding a trigger for pain, or participating in the treatment for the underlying pain condition. In this study, we have focused on one particular aspect of pain anticipation – the avoidance of the stimulus, which can be reliably identified in rodent models. Future studies are thus needed to examine other aspects of pain anticipation. However, based on existing human imaging data on the role of ACC in pain anticipation, it is likely that some of the coding mechanisms identified in this study can be applied to these other aspects of pain anticipation as well.

In conclusion, after a period of conditioning, neurons in the ACC can progressively anticipate pain. Thus, our results provide clues into disorders in pain anticipation and its avoidance such as catastrophization, and offer insight into effective coping strategies, especially strategies such as cognitive behavioral therapy that engage higher cortical functions.

## Materials and Methods

### Animals

All procedures were approved by the New York University School of Medicine (NYUSOM) Institutional Animal Care and Use Committee (IACUC) as consistent with the National Institute of Health (NIH) *Guide for the Care and Use of Laboratory Animals* to ensure minimal animal use and discomfort. Male Sprague-Dawley rats were purchased from Taconic Farms, Albany, NY and kept at Mispro Biotech Services Facility in the Alexandria Center for Life Science, with controlled humidity, temperature, and 12 hr light-dark cycle. Food and water were available *ad libitum*. Animals arrived to the animal facility at 7 weeks and were given on average 10 days to adjust prior to the onset of experiments.

### Behavior experiment

During the pre-conditioning phase of 30 trials, a tone stimulus (4 KHz, 80 dB) was paired with a non-noxious thermal stimulation (NS, 50 mW) applied to the plantar surface of the hind paw contralateral to the brain recording site, in freely moving rats. The non-noxious stimulus was applied to the hind paws for 10 seconds. In the majority of cases, there weren’t withdrawals in response to NS, referred to as “immobile” trial. (We choose the terminology “immobile” rather than “freezing” as the animals didn’t exhibit a classical freezing behavior as described notably in tone/foot shock pairing experiment). Right after, during the conditioning phase of 50 trials, the same tone stimulus was paired with noxious thermal stimulation (HS, 250 mW) applied to the hind paw for 5 seconds or until the animals withdrew. If the animal moved after the tone was played (−3 seconds), and before the laser was turned ON (0 second), the trial was counted as an avoided trial. In this case, the latency of withdraw was quantified as a negative value in regard to the time of laser ON, and the painful stimulus was not applied to the hindpaw of the rat. In the case when an animal did not move before the laser turns ON, the stimulus was applied to the hind paw for 5 seconds or until the animal withdrew. The latency of withdrawal was quantified as a positive value in regard to the time of laser ON and was referred to as withdrawal trial. Accordingly, each trial of each animal was quantified by 2 factors: time of withdrawal (Fig. 1c,d,g,h) and identity as an avoiding or withdrawal trial (Fig. 1e,f). The 2 factors are linked as an avoiding trial is quantified by a negative value of withdrawal latency; a withdrawal trial is quantified by a positive value. Then, for each animal, we calculated its withdrawal latency over time. An animal with a decrease in latency over time as demonstrated by an inverse linear relationship with the number of trials was considered an avoider. An animal with an increase in withdrawal latency over time as demonstrated by an positive linear relationship with the number of trials was considered a non-avoider. We represented the average for all animals constituting a group.

Finally the animals underwent a post-conditioning phase of 30 trials, similar to the pre-conditioning phase. Inter-trial intervals were 30–60 sec in all phases (). A video camera (HC-V550, Panasonic) was used to record the experiment and to quantify the withdraw latency.

### Electrode implant and surgery

Tetrodes were constructed from four twisted 12.7 µm polyimide-coated microwires (Sandvik) and mounted in an eight tetrode VersaDrive (Neuralynx). Electrode tips were plated with gold to reduce electrode impedances to 100–500 kΩ at 1 kHz. Rats were anesthetized with isoflurane (1.5–2%). In aseptic conditions, rats were mounted in a stereotaxic apparatus. The scalp was incised and a 3-mm-diameter hole was drilled in the skull, above the anterior cingulate cortex. A durotomy was performed before tetrodes were slowly lowered unilaterally into the ACC (AP +2.8 mm, ML +0.8 mm, and DV −1.5 mm, with tetrode tips angled 5° toward the midline). The drive was secured to the skull with screws and dental cement.

After animal sacrifice, brain sections (20 µm) were collected using a Leica M3035 Cryostat machine, and histologically stained for tetrode localization. Animals with improper electrode placements were excluded from further analysis (none in this case).

### Immunohistochemistry

Rats were deeply anesthetized with Isoflurane (5% for 10 min) and transcardially perfused with PBS. Brains were fixed in PFA (4%) for 24 h and then transferred to 30% sucrose in PBS for three days. 20–30 µm coronal sections were stained with cresyl violet, washed in PBS and coverslipped with Vectashield mounting medium then viewed and recorded under a Nikon eclipse 80i microscope with a DS-U2 camera head.

### Neural data collection and preprocessing

Tetrodes were lowered in steps of 60 µm before each day of recording (2 sessions per animals). The neuronal activity and the onset of tone and laser stimulation were simultaneously recorded with acquisition equipment (Open Ephys) via an RHD2132 amplifier board (Intan Technologies). Signals were monitored and recorded from 32 low-noise amplifier channels at 30 kHz, band-passed filtered (0.3 to 7.5 kHz). To get spike activity, the raw data were high-pass filtered at 300 Hz with subsequent thresholding and offline sorting by commercial software (Offline Sorter, Plexon). The features of three valley electrodes were used for spike sorting. The reliability of cluster separation was verified by inspecting cross-correlograms. Trials were aligned to the initiation of the peripheral stimulus to compute the PSTH for each single unit using MATLAB.

For neuronal spike analysis, to define a neuron that altered its firing rate in response to a peripheral stimulus, we calculated peri-stimulus time histograms (PSTH), using a 10 s range before and after laser stimulus and a bin size of 250 ms. The baseline mean and standard deviation was calculated from a 3 second interval prior to the tone. To calculate Z-scored firing rate (FR), we used the following equation:

1 $${\rm{Z}} \mbox{-} \mathrm{scored}\,{\rm{FR}}=({\rm{FR}}-{\rm{mean}}\,{\rm{of}}\,{{\rm{FR}}}_{{\rm{b}}})/{\rm{standard}}\,{\rm{deviation}}\,({\rm{SD}})\,{\rm{of}}\,{{\rm{FR}}}_{{\rm{b}}},$$

where FR_b_ indicates the baseline firing rate prior to the tone. To define a pain responsive neuron, we used the following criteria: (1) The absolute value of the Z-score firing rate of at least one time bin after stimulation must be ≥2.5; and (2) If the first criterion is passed, at least the next two bins must be greater than 1.65. Lastly, these criteria must be fulfilled in the time window analyzed seconds after the stimulus.

### Model-based approach for change-point detection

We have developed two statistical (model-based vs. model-free) approaches for detecting abrupt changes in neural ensemble activity based on rat ACC ensemble recordings.

*Notations*: Given spike train recordings of *C* units, let ***y***_*k*_ = [*y*_1,*k*_, …, *y*_*C*,*k*_] denote a *C*-dimensional population vector, with each element consisting of the binned neuronal spike count at the *k*-th time bin (with bin size ∆); let exp(***η***_*k*_) denote the Poisson firing rate vector for *C* neurons.

We considered a latent variable model that links the neural activity to an external stimulus (e.g., pain stimulus). We assumed that the population spike data follows a latent-state Poisson linear dynamical system (PLDS), where the univariate variable *z*_*k*_ denotes an unobserved common input that drives neuronal ensemble spiking activity ***y***_*k*_ as follows:

2 $${z}_{k}=a{z}_{k-1}+{\epsilon }_{k}$$

3 $${{\boldsymbol{\eta }}}_{k}={\bf{c}}{z}_{k}+{\bf{d}},$$

4 $${{\boldsymbol{y}}}_{k} \sim Poisson\,(\exp ({{\boldsymbol{\eta }}}_{k}){\rm{\Delta }})$$

where the state equation (2) is a first-order autoregressive (AR) model (0 < |*a*| < 1) driven by a Gaussian noise process with zero mean, and variance *σ*^2^. We used an iterative expectation-maximization (EM) algorithm to estimate the latent state sequence *z*_1:*T*_ (E-step) and the unknown parameters {*a*, **c**, **d**, *σ*^2^} (M-step). For mathematical details, refer to previous publications^18,65,66^.

From the inferred latent variable sequence, we computed the Z-score related to the baseline period^18^

5 $${\rm{Zscore}}=\frac{{z}_{k}-{\rm{mean}}\,{\rm{of}}\,{z}_{{\rm{baseline}}}}{{\rm{SD}}\,{\rm{of}}\,{z}_{{\rm{baseline}}}}$$

and further converted it to probability

6 $$P({\rm{Zscore}} > \theta )=1-{\int }_{-\infty }^{\theta }\frac{1}{\sqrt{2\pi }}\exp \,(-\frac{{u}^{2}}{2})du$$

The criterion of a significant change in Z-score is determined by a critical threshold θ > 0 based on the 95% significance level. Specifically, let CI denote the confidence interval (CI) derived from the standard deviation of the latent variable, it is concluded when Z-score-CI > θ or Z-score + CI < −θ. Here we set the threshold θ = 1.65. Note that the algebraic sign of the Z-score is irrelevant due to the sign ambiguity of the latent variable in model identification.

### Model-free approach for change-point detection

We designed a greedy CUSUM (cumulative sum) algorithm to detect the change in Poisson firing rates. The CUSUM method is aimed to detect a change point based on the instantaneous log-likelihood ratio (LLR)^65^:

7 $${{\rm{LLR}}}_{{f}_{1}||{f}_{0}}=y\,\mathrm{log}\,\frac{{\lambda }_{1}}{{\lambda }_{0}}-({\lambda }_{1}-{\lambda }_{0})$$

where *y* denotes the spike count observation, *λ*_0_ and *λ*_1_ are the rate parameters of two Poisson distributions *f*_0_ and *f*_1_. We assumed that the baseline rate *λ*_0_ is known or can be estimated directly from the baseline spike data. We defined an empirical criterion for a significant change in *λ*_1_ such that ${\lambda }_{1}^{sig}={\lambda }_{0}+3\sqrt{{\lambda }_{0}}$ in a positive direction. The CUSUM algorithm computed the instantaneous LLR *s*_*c,k*_ for the *c*-th neuron and updated the cumulative sum from *c* = 1, …. *C*

8 $${S}_{k}={{\rm{\max }}}_{c}\{{S}_{c,k}\}={{\rm{\max }}}_{c}\{\,{\rm{\max }}\,\{0,{S}_{c,k-1}+{s}_{c,k}\}\}$$

where *S*_0_ = 0. When the cumulative sum *S*_*k*_ was above a predetermined threshold *θ*_0_ and the trend continued more than a few consecutive steps (~150 ms) in a monotonic manner, we reached a decision on the change point. The threshold *θ*_0_ in the CUSUM algorithm controls the false alarm rate. An empirical choice is to use the test statistic (twofold log-likelihood), being a chi-square distribution with 1 degree of freedom: ${\chi }_{1,(1-\alpha )}^{2}$. We set *α* = 0.01 such that *θ*_0 = _0.5 × 6.64 = 3.38.

In our data analysis, we applied both model-based and model-free methods to detect the abrupt changes of ACC neuronal ensemble spiking activity in relation to the baseline.

### Statistical analysis

The results of behavioral experiments were given as mean ± SEM. An unpaired Student’s t test was used to compare the withdrawal latency of each animals group during each phase. Two-way ANOVA, and post-hoc Bonferroni t-test was used to compare the 2 groups of animals during the conditioning phase and pre/post conditioning phases. A Chi square test was performed to compare the distribution of the behavior responses. Fisher’s exact test was performed to compare the neuronal population distribution.

All neuronal firing rates had a non-normal distribution. Thus, non-parametric tests were performed. For unpaired data, the Mann-Whitney U-test (rank-sum test) was performed to test the equivalence of distributions. For paired data, the Wilcoxon matched-pairs signed rank test was used to test the equivalence of distributions.

For all statistical tests, a *p* value < 0.05 was considered statistically significant. All data were analyzed using the GraphPad Prism Version 7 software (GraphPad) and MATLAB (MathWorks).

## Electronic supplementary material

Supplementary figures
