# Supplementary material for: Rate and Temporal Coding Mechanisms in the Anterior Cingulate Cortex for Pain Anticipation
Source: Sci Rep. 2018 May 29;8:8298. doi: 10.1038/s41598-018-26518-x (PMC5974274; doi:10.1038/s41598-018-26518-x)
Supplement: Supplementary file 1 — Supplementary figures [file 41598_2018_26518_MOESM1_ESM.pdf]

Supplementary information

Rate and Temporal Coding Mechanisms in the Anterior Cingulate Cortex for Pain Anticipation

Louise Urien[1], Zhengdong Xiao[2,5], Jahrane Dale[1], Elizabeth P. Bauer[4], Zhe Chen[2,3], and Jing Wang[1,3*]

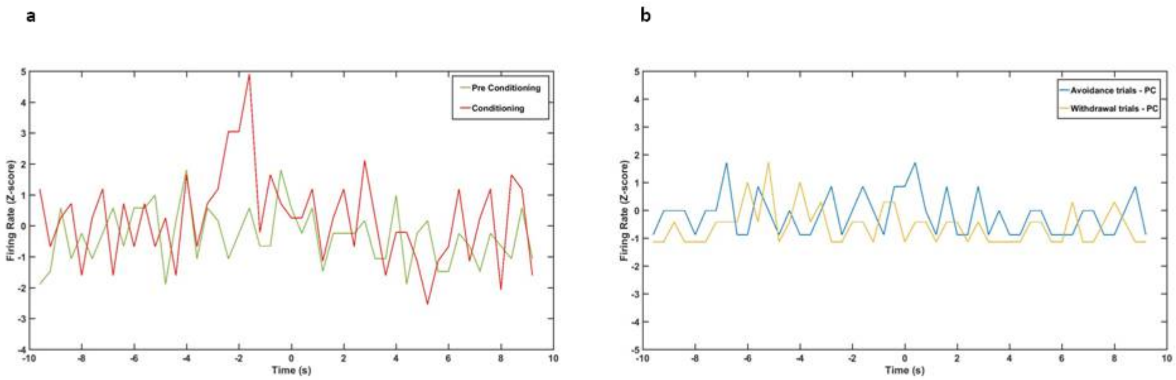

Supplementary Figure 1. Specificity of "pain-anticipating neurons"

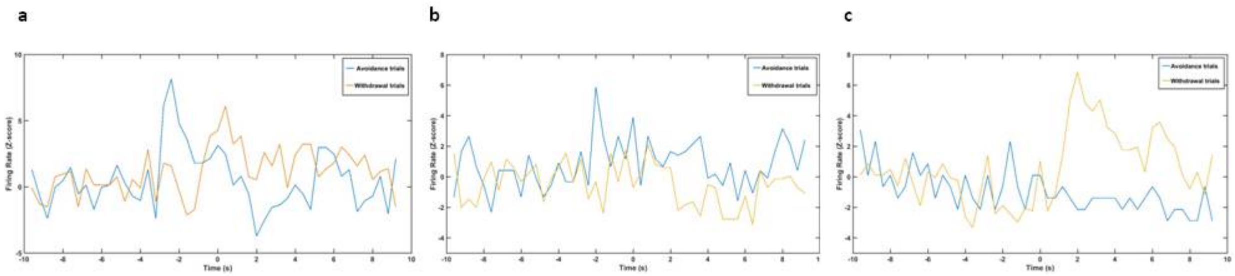

Supplementary Figure 2. Example of neurons differentially firing during avoided versus withdrawal trials

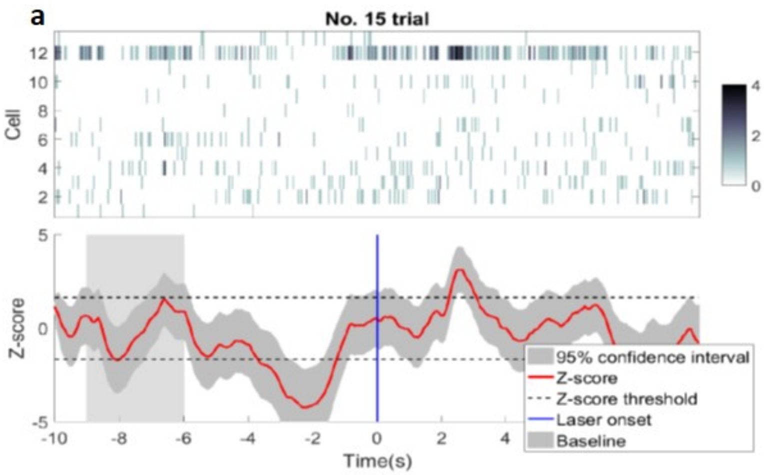

Supplementary Figure 3. Unsupervised learning method detects a change point in population spike activity when the animal avoids a painful stimulus

Figure legends

Supplementary Figure 1. Specificity of "pain-anticipating neurons"

(a) Firing rate of a representative neuron that increases its firing rate during the anticipatory period during conditioning (red line) but not during pre conditioning (green line), we called those neurons "pain-anticipating" neurons. Time = 0 corresponds to the time of the onset of the pain stimulus. (b) The same "pain-anticipating" neurons do not change its firing during the pre conditioning phase when the animal moves, avoided trials (blue line), withdrawal trials (yellow line).

Supplementary Figure 2. Example of neurons differentially firing during avoided versus withdrawal trials

Firing rate (Z-score) of a 3 neurons (a) Example neuron increases its firing rate during the anticipatory period (-3 to 0 second) during the avoided trials (blue line) but does not change its firing rate during the withdrawal trials (yellow line). (b) Example neuron does change its firing rate during the anticipatory period during the avoided trials (blue line) but increases the spike rate after

the painful stimulus during the withdrawal trials (yellow line). (c) Example neuron does not change its firing rate during the anticipatory period during the avoided trials (blue line) but increases the spike rate after the painful stimulus during the withdrawal trials (yellow line). Time = 0 corresponds to the time of the onset of the pain stimulus.

Supplementary Figure 3. Unsupervised learning method detects a change point in population spike activity when the animal avoids a painful stimulus

Example of single-trial decoding analysis in an ACC neuronal population (13 units) during an avoided trial. Sorted population spike counts (top panel). Bin size 50 ms. Color bar indicates spike count, with the dark color representing high spike count. Bottom panel: estimated mean Z-score (red curve) from the univariate latent state. The shaded area marks the confidence intervals. Baseline is calculated from -9 to -6 sec (grey bar). The vertical blue lines indicate the laser onset (time= 0 sec). Horizontal dashed lines mark the thresholds of the significant zone. The model-based method (Materials and Methods) can detect a change in population spike activity ~2 sec before laser onset, at the time when the animal avoids the laser stimulus.
